# Interferometric Reflectance Imaging Sensor (IRIS)—A Platform Technology for Multiplexed Diagnostics and Digital Detection

**DOI:** 10.3390/s150717649

**Published:** 2015-07-20

**Authors:** Oguzhan Avci, Nese Lortlar Ünlü, Ayça Yalçın Özkumur, M. Selim Ünlü

**Affiliations:** 1 Department of Electrical and Computer Engineering, Boston University, 8 St. Mary’s Street, Boston, MA 02215, USA; E-Mail: oguzhan@bu.edu; 2 Department of Biomedical Engineering, Boston University, 8 St. Mary’s Street, Boston, MA 02215, USA; E-Mail: nese.lortlar@gmail.com; 3 Department of Electrical and Electronics Engineering and School of Medicine, Bahcesehir University, Ciragan Caddesi, No:4-6, Besiktas, Istanbul 34353, Turkey; E-Mail: aozkumur@gmail.com

**Keywords:** optical biosensor, interferometry, label-free, digital detection, nanoparticle, virus, single nucleotide polymorphism

## Abstract

Over the last decade, the growing need in disease diagnostics has stimulated rapid development of new technologies with unprecedented capabilities. Recent emerging infectious diseases and epidemics have revealed the shortcomings of existing diagnostics tools, and the necessity for further improvements. Optical biosensors can lay the foundations for future generation diagnostics by providing means to detect biomarkers in a highly sensitive, specific, quantitative and multiplexed fashion. Here, we review an optical sensing technology, Interferometric Reflectance Imaging Sensor (IRIS), and the relevant features of this multifunctional platform for quantitative, label-free and dynamic detection. We discuss two distinct modalities for IRIS: (i) low-magnification (ensemble biomolecular mass measurements) and (ii) high-magnification (digital detection of individual nanoparticles) along with their applications, including label-free detection of multiplexed protein chips, measurement of single nucleotide polymorphism, quantification of transcription factor DNA binding, and high sensitivity digital sensing and characterization of nanoparticles and viruses.

## 1. Background and Motivation

Diagnostics and understanding of the etiology of the disease have been a focus of medical and biotechnology research for many years. Disease diagnostics have been evolving through the synergistic collaboration of medicine with engineering and science. Until the 20th century, clinical diagnostics relied mostly on medical history and physical examination. Clinical tests actually date back thousands of years to the time of Hippocrates, when the color and odor of urine was utilized in diagnostics. With the advent of the measurement/sensing technologies that provided the ability of detecting trace substances in bodily fluids, such as blood, urine, and cerebrospinal fluid, *in vitro* tests have become a cornerstone of clinical practice. These tests range from bacterial cultures to DNA chips for genetic profiling and from lateral flow tests to protein microarrays. Despite dramatic technological advances over the last decade, recent emerging infectious diseases and epidemics have exposed the limitations of current technologies and once again emphasized the importance of continuing innovation and refinement.

Various modalities of biosensing have been applied to detection of biological markers. These biomarkers play a critical role both in healthy physiological conditions and during the course of diseases that threaten human health, such as cancer, cardiovascular diseases, infectious diseases, neurologic diseases, and many others. Depending on the application, diagnostics can rely on detection of biomarkers related to infectious agents (such as viruses, bacteria, yeasts, *etc.*) and toxins, as well as markers related to host immune response and changes in the physiological conditions. In recent years, the ability to detect biomarkers in extremely low concentrations has led to advances in basic and clinical research and in their predictive role regarding diagnosis, prognosis, and progression of diseases. Highly sensitive, specific, quantitative, and multiplexed detection of biomarkers will be a pivotal focus of the technological evolution of future generation diagnostics [1].

A variety of transduction and amplification mechanisms, such as electrochemical, mechanical or optical techniques, have been used for detection of serum biomarkers [2,3]. Here we focus on an optical biosensor technology that relies on label-free interferometric sensing. Direct monitoring of primary molecular binding interactions, without the need for secondary reactants, would markedly simplify and expand applications of high-throughput, label-free detection methods. We have developed the Interferometric Reflectance Imaging Sensor (IRIS) for label-free, high-throughput, high-sensitivity, and dynamic detection of molecular binding on a solid surface, and demonstrated sensing of protein-protein binding and DNA-protein binding in a real time, with high sensitivity (~10 pg/mm^2^) and reproducibility [4,5], as well as label-free measurements of DNA hybridization kinetics [6] and virus detection [7]. Recent significant advancements in IRIS technology have allowed us to identify individual captured nanoparticles based on size and shape [8,9]. This new modality of IRIS is termed single-particle IRIS (SP-IRIS) and it allows for high-sensitivity virus detection [10]. In this article, we review the operating principles of IRIS and its relevant features for quantitative, label-free, and dynamic detection. We also present the two different modalities for IRIS: (i) low-magnification (ensemble biomolecular mass measurements) and (ii) high-magnification (digital detection of individual nanoparticles and viruses) along with their applications.

## 2. Interferometric Reflectance Imaging Sensor (IRIS)

The IRIS signal is based upon the interference of the fields reflected off a layered substrate that is typically comprised of a SiO_2_ layer thermally grown atop a Si surface. As previously indicated, IRIS renders two distinct detection modalities: (i) high-throughput label-free measurement of biomass accumulations; and (ii) digital detection of single particles with high-magnification, also known as Single Particle Interferometric Reflectance Imaging Sensor (SP-IRIS). Both modalities are described in detail in the following sections.

### 2.1. High-Throughput Label-Free Biomass Detection with IRIS

**Figure 1:**
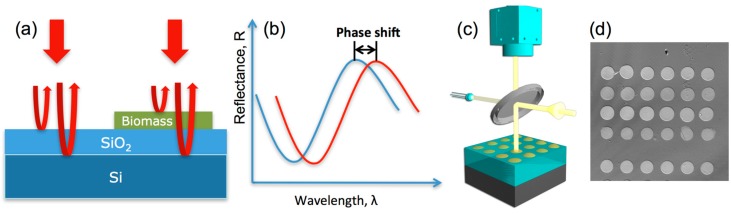
(**a**) Schematics of the working principle of IRIS showing the interference of light reflected from the reference plane (Si-SiO_2_ interface) with that from the top surface. (**b**) Resulting shift in the wavelength dependent reflectance characteristics due to added biomass. A simple optical imaging system (**c**) using either continuously tunable or discrete wavelength sources can be used to determine the surface profile as shown in a typical IRIS image of protein spots (**d**) allowing for measuring molecular binding for thousands of spots.

In this biosensing modality of IRIS, transduction is based on spectral reflectivity. As the overall thickness of the upper layer is increased due to biomass accumulation on the surface of the layered substrate, the optical path difference (OPD) between top surface of the substrate and the Si-SiO_2_ interface also increases, which in turn results in a quantifiable shift in spectral reflectivity (Figure 1). The reflectivity curves are sampled at different wavelengths by a CCD and fitted to spectral reflectance signatures using Fresnel equations given below:

$$R={\left|r\right|}^{2}=\frac{r_{1}^{2}+r_{2}^{2}+2r_{1}r_{2}\cos2\phi }{1+r_{1}^{2}+r_{2}^{2}+2r_{1}r_{2}\cos2\phi }$$

$$\text{where}\;r_{1}=\frac{n_{2}-n_{1}}{n_{2}+n_{1}},\;r_{2}=\frac{n_{3}-n_{2}}{n_{3}+n_{2}}\;\text{and}\;\phi =\frac{2\text{π}d}{\text{λ}}n_{2}\cos\text{θ}$$

where *r*_1_ and *r*_2_ denote the Fresnel reflection coefficients, *n*_1_, *n*_2_, and *n*_3_ are the indices of refraction for air, SiO_2_, and Si, respectively. λ denotes the wavelength of incident light and θ denotes the angle of incidence. As the optical system uses a low-numerical aperture (NA) objective (~0.1, *i.e.*, θ <6°), the polarization dependence of the incident light is negligible.

Notice that monitoring the incident light is crucial in order to accurately obtain the reflectivity curves from a layered substrate by accounting for any fluctuations in the incident light. Earlier versions of IRIS included a photodetector for this purpose, yet a more elegant and simpler technique has been implemented in recent versions, where, instead of an additional detector element, a reference region on the sensor chip surface has been designated to normalize any fluctuations in the incident light [11]. The details of the evolution of the IRIS platform will be provided in the Instrumentation section.

Low magnification modality of IRIS provides label-free, quantitative, dynamic, and multiplexed monitoring of biomolecular binding on the sensor surface. Applications of this modality of IRIS include precise quantification of DNA and protein adsorption, real-time monitoring of binding events, and accurately measuring the association and disassociation rates. The studies detailed in the Results section are critically dependent on these features of the IRIS platform.

### 2.2. Digital Detection of Single Particles with SP-IRIS

**Figure 2:**
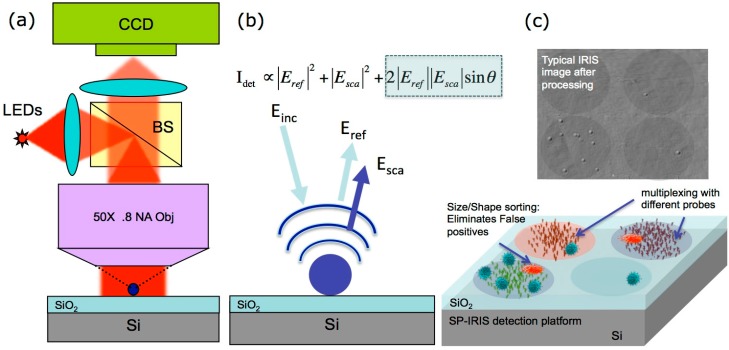
(**a**) In SP-IRIS, visible LED provides illumination, and bright field reflection image is captured on a CCD camera; (**b**) The key to improved visibility of nanoparticles on the SP-IRIS system is the mixing of scattered light with reference light reflected from the Si-SiO_2_ interface. The cross term (highlighted) can be orders of magnitude larger than the scattered intensity (middle term) for small low-index nanoparticles. Thus when illuminated by a narrow spectrum source (LED), surface bound nanoparticles (e.g., viruses) become visible; (**c**) A typical SP-IRIS chip with multiplexed capture antibodies. Detected particles appear as diffraction-limited dots in the image and the contrast of the dots can be correlated to their sizes, thus allowing for discrimination and elimination of noise in complex target solutions. (Reused with permission from [12]).

Single Particle IRIS (SP-IRIS) has been adapted from the previously described modality of IRIS. Essentially, it extends the wide-field interferometric imaging to high spatial resolution using a high-NA objective. In this case, the signal is based upon the interference between the scattered field from the particle of interest and the reference field reflected off the interface of the layered substrate as illustrated in Figure 2 [12].

The thickness of the SiO_2_ layer determines the phase shift introduced between the scattered and reflected reference fields, and in SP-IRIS, it is tuned to optimize the signal (contrast) for a given particle, enabling highly sensitive detection and counting of nanoscale particles. Furthermore, the SP-IRIS signal is primarily affected by the polarizability of the particle (α), amplitude of the reference field (E*_ref_*) and the phase lag between them (θ) as given in the following equations:

$$\text{α}=4\text{π}\epsilon_{o}r^{3}\frac{\epsilon_{p}-\epsilon_{m}}{\epsilon_{p}+2\epsilon_{m}}$$

$${\left|E_{sca}\right|}^{2}\propto \;{\text{α}}^{2}$$

$$I\;\propto {\left|E_{ref}\right|}^{2}+{\left|E_{sca}\right|}^{2}+2\left|E_{ref}\right|\left|E_{sca}\right|cos\text{θ}$$

In this equation, for small particles, ${\left|E_{sca}\right|}^{2}\;$can be neglected, and thus the cross term ($\left|E_{ref}\right|\left|E_{sca}\right|$) dominates the signal from the nanoparticles in the SP-IRIS image. In other words, due to interference, light reflected from the sensor surface is modified by the presence of particles producing a distinct signal that is captured by a camera. This signal appears as a dot in the image and, with a forward model, the size of the particle is calculated based on its contrast in the image. In particular, particle sizing provides a means to discriminate target particles from impurities such as dust particles and aggregates. As the diameters of the particles of interest are much less than the illumination wavelength, the forward model assumes the particles to be Rayleigh scatterers, and uses angular spectrum representation [13] to obtain the image of the particles in far-field for a wide-field interferometric imaging scheme. In this manner, the contrast of the diffraction-limited spot in the image is related to the physical size of the particle if the optical properties are known *a priori* [8,9]. In addition, the signal can be tuned by particle geometry, substrate type, illumination wavelength, and defocus. Recent efforts to improve SP-IRIS signal included incorporating polarization optics into the system to achieve an enhanced signal using metallic particles such as gold nanorods and gold nanospheres. With the aid of polarization optics, the reference field can be effectively reduced, extending IRIS platform’s single particle detection modality to a partial dark-field interferometric imaging scheme that allows for significant contrast enhancements for the nanoparticles.

The use of functionalized nanoparticles for protein detection promises very high sensitivity along with quantitative detection, allowing for single particle/molecule counting. IRIS technology’s capability exceeds the state-of-the-art detection systems because it provides single molecule sensitivity with non-complex and cost-effective constituents: a simple sensor substrate, LEDs, an optical setup with conventional optics, and a CCD detector.

## 3. Instrumentation

**Figure 3:**
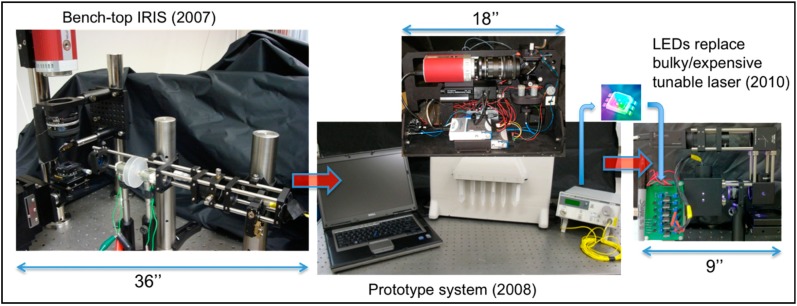
Early evolution of IRIS: (**left**) First generation IRIS setup which used a tunable laser and spanned half of an optical table; (**center**) A first generation prototype produced by Zoiray Technologies; (**right**) A second generation IRIS prototype with multi-color LED sources.

IRIS has gone through several stages of commercializable biosensor prototype development since its first design in 2007 on an optical bench using a continuously tunable laser source (Figure 3). Within a year, a prototype instrument (with a tunable laser) was commercialized by Zoiray Technologies for protein microarrays. Starting in 2010, we have replaced the bulky/expensive tunable laser with discrete LED sources [14] and reduced the size, cost, complexity, and power requirements of the instrument. The evolution of the IRIS and SP-IRIS instrumentation is described in detail in [15]. For the two distinct modalities of operation, the significant difference of IRIS instruments is the magnification of the optical system or the numerical aperture of the objective lens. While it is possible to combine both modalities in one instrument, utilizing multiple objectives similar to an upright optical microscope with a turret, it is often more desirable to optimize other parameters of the overall system for either label-free, low-magnification IRIS or the high-magnification SP-IRIS. As shown in Figure 3, a low-magnification IRIS system has been reduced in complexity and size after LEDs replaced the tunable laser and can be further miniaturized. One of the important performance parameters is the field of view (FOV), which can be improved with the advent of camera technology. For example, we have demonstrated an IRIS system utilizing a consumer product digital camera with a field of view of approximately 1 inch-square.

**Figure 4:**
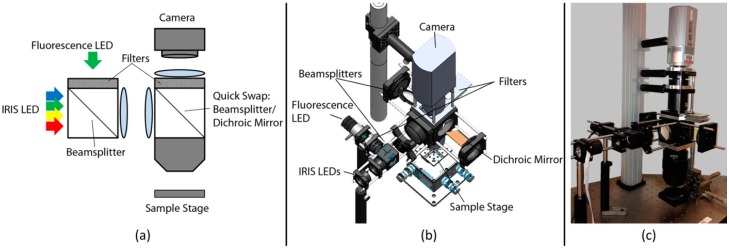
CaFE instrument design. (**a**) Schematic of the optical layout. For fluorescence measurements, the second beamsplitter is swapped for a dichroic mirror and an emission filter is inserted; (**b**) Instrument model with main components indicated; (**c**) Image of the actual instrument. (Reused with permission from [16]).

Since IRIS allows for accurate and label-free quantitation of the surface bound biomolecules, it can be used for quality control of bioassay chips. With a 1 inch-square field of view, biomolecule spots on a microarray prepared on an IRIS chip conforming to the size of a microscope slide (25 mm × 75 mm) can be read and quantified by taking three images in less than one minute. It is also possible to combine label-free calibration with standard fluorescence reading in a single instrument. A hybrid prototype that calibrates fluorescence measurements with IRIS mass density measurements has been introduced to address the innate problem associated with the variations in probe immobilization that can often result in false negatives in immunodiagnostics [16]. The technique is termed calibrated florescence enhancement (CaFE) and uses a Si-SiO_2_ layered sensor chip, which provides tunable enhancement in fluorescence emission compared to glass substrates that are usually used in fluorescence microarrays [17]. From an optical design standpoint, IRIS and wide-field fluorescence imaging have several similarities and two significant differences. Both platforms are top-illuminated, also known as reflection-mode, microscopes. For reliable data collection, these instruments require uniform and stable illumination of the sample across the FOV. The differences stem from fluorescence detection necessitating spectral filters and a high NA collection. Taking into account the requirements of the modalities, the hybrid instrument has been designed as shown in Figure 4.

Due to the high optical magnification, SP-IRIS system requirements are more stringent. For this modality, a fully automated prototype allowing for automated focusing and sample scanning, and subsequent data analysis has been developed. For this technology to move forward as a diagnostic platform, the dependence on the user’s focusing skills must be removed. To this end, an automated and easy-to-use SP-IRIS has been designed, constructed, and tested. Two complimentary focusing algorithms are implemented in tandem to achieve the ±50 nm accuracy for robust nanoparticle detection [14,18]. SP-IRIS technology has been later commercialized by a spin-off from Boston University (NexGen Arrays, Inc., Boston, MA, USA).

## 4. Applications and Results

The multifunctional IRIS platform has been extensively utilized in various studies, with results described below. As a label-free and quantitative technique, IRIS enables multiplexed protein detection. Its dynamic measurement capability allows for single nucleotide polymorphism (SNP) determination by DNA melting, and its absolute quantification capability allows for development of calibration as well as fundamental studies of transcription factor—DNA binding. Moreover, its digital sensing modality (SP-IRIS) allows for high sensitivity virus detection and identification. We review examples of these applications below.

### 4.1. Label-Free Detection with Protein Microarrays

**Figure 5:**
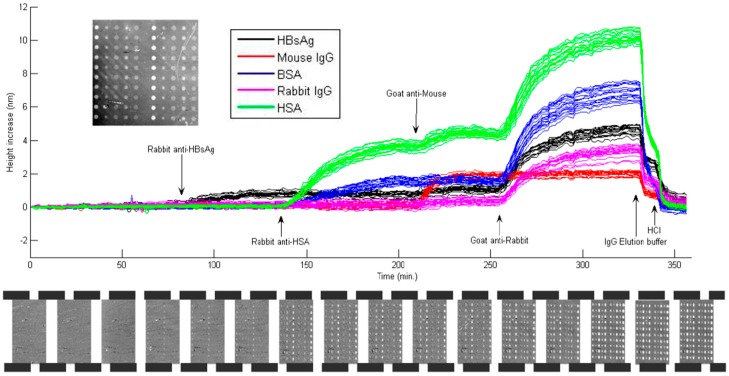
Antibody capture experiment with several layers of interacting antigens and antibodies. Upon incubation with anti-HBsAg, anti-HSA, anti-mouse and anti-rabbit antibodies, specific capture of these antibodies by the corresponding antigens is observed. Bottom panel shows snapshots of representative IRIS images. (Reused with permission from [4]).

IRIS platform’s label-free mass density measurement modality has been a powerful tool for applications that require dynamic monitoring of bimolecular interactions to investigate events such as disease progression and binding kinetics; and protein microarrays have played a significant role in this modality’s development by providing target-specific immunoassays. The utility of this technique has been demonstrated in [4], with a particular focus on the dynamic measurements of antibody-antigen interactions. The study uses four different protein probes, bovine serum albumin (BSA), human serum albumin (HSA), rabbit IgG and protein G, immobilized as multiple spots on an IRIS chip functionalized with an epoxysilane coating. As seen from the results shown in Figure 5, upon reaction with a specific antigen, each spot shows a height increase observed over time, allowing for the measurements of kinetic association and dissociation rates. A dilution experiment demonstrates a limit of detection (LOD) of less than 20 ng/mL corresponding to approximately 120 pM. The LOD for the low-magnification, label-free modality of IRIS is calculated from 1.96 times the standard deviation divided by the slope of the binding curve in linear region as explained in [4].

Label-free multiplexed virus detection has also been demonstrated on IRIS using serum targets containing vesicular stomatitis virus (VSV), as it conveniently offers versatility in terms of modification of surface proteins for surrogate detection of hemorrhagic fever viruses (such as Ebola and Marburg) whilst maintaining a biosafety level-2 (BSL2) operation [7]. A limit of detection down to 3.5 × 10^5^ plaque-forming units/mL (PFU/mL) is confirmed for specific virus detection with a simple surface chemistry and minimal sample preparation.

### 4.2. Label-Free Detection with DNA Microarrays

**Figure 6:**
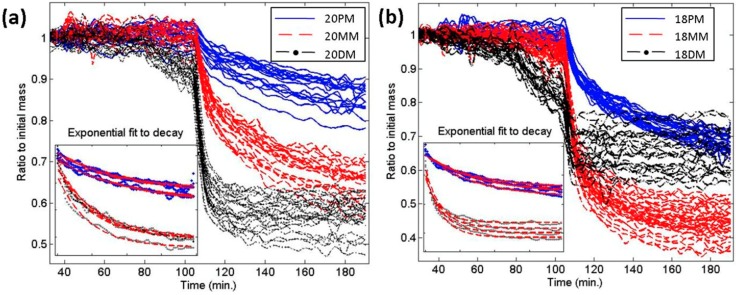
Kinetic characterization of DNA dissociation. (**a**) Dynamic dissociation graphs of 20 mers; (**b**) Dynamic dissociation graphs of 18-mers. Single decaying exponentials were fit to each of these curves for the time period between 110th and 180th minutes, to find the decay constants (shown in the inset plots). (Reused with permission from [6]).

DNA microarrays have gained significant attention in medicine because of their aptitude for multiplexed detection of nucleotides and are utilized in a wide variety of applications from gene expression profiling to diagnostics. IRIS allows for dynamic label-free quantitation of surface bindings and has been deployed for single nucleotide polymorphism (SNP) detection in a DNA microarray format in [6]. The study explores the denaturation kinetics and its ionic concentration dependency. Dynamic measurements of DNA mass density were carried out over time and DNA denaturation kinetics for duplexes with perfect match (PM), single mismatch (SM), and double mismatch (DM), as well as a negative control single stranded DNA probe were observed. The mismatch detection was based upon denaturation kinetics measured by the change in the mass density of a spot over time for 20-basepair PM (20PM), 20MM and 20DM spots. The results (shown in Figure 6) indicate easily distinguishable denaturation rates for different types of mismatches.

Moreover, to further quantify the denaturation rates, the measured data was fit to an exponential function as shown in Figure 6. The denaturation rates differ significantly, and IRIS is capable of detecting SNP with the following confidence intervals obtained from a single spot: 98.6% for 18-mer single mismatch detection, 97.2% for 20-mer single mismatch detection, and 88.7% for distinguishing 18PM from 20PM. The authors also note that those confidence intervals can be improved beyond 99% by simply using multiple spots and averaging the data [6].

### 4.3. Protein Detection with DNA Microarrays

Transcription in eukaryotic cells is initiated by the interactions between a specific type of protein called transcription factor (TF) and the promoter region of DNA making up what is known as the preinitiation complex (PIC). A deeper understanding of these interactions involving TFs can shed light on the eukaryotic transcription initiation processes, hence the resultant gene expression regulations. With this goal in mind, IRIS has been used to study those interactions in [19]. Specifically, the study explores TATA binding protein (TBP), a key TF that takes part in the initiation of eukaryotic transcription. The results indicate high amounts of binding of TBP to both single stranded (ss) and double stranded (ds) DNA with a TATA motif, as well as to ss25-mer T. TBP to DNA binding ratio for ssDNA and dsDNA with a TATA motif is ~1, whereas this ratio goes up to ~4 for ss25-mer T suggesting that multiple TBPs bound to a single ss25-mer T. A computational genome analysis revealed that poly-T stretches are abundant (35.5%) in promoter regions. Based on the positional analysis of poly-T stretches and TATA box, it is concluded that there are 51 TATA-less promoters that can be regulated by TBP through the presence of poly-T stretch in the core promoter.

IRIS has also been used to study the ferric uptake regulatory protein (Fur)—a transcriptional regulatory protein that functions to control gene transcription in response to iron in a number of pathogenic bacteria. Using the high-throughput capability of IRIS, Fur-DNA interactions were characterized *in vitro* with predicted Fur binding sequences in the genome of *Neisseria gonorrhoeae*, the causative agent of the sexually-transmitted disease gonorrhea. These studies demonstrate that 70% of the predicted Fur boxes in promoter regions of iron-induced genes bound to Fur, *in vitro*, with a range of affinities as observed using this microarray screening technology. Combining binding data with mRNA expression levels in a gonococcal Fur mutant strain allowed for the identification of five new gonococcal genes under Fur-mediated direct regulation [20].

### 4.4. Microarray Quality Control and Calibrated Fluorescence Enhancement (CaFE)

Spot-to-spot and chip-to-chip variability in microarray technology is an essential concern in producing reliable data, not only due to technical variations, such as array printing, sample processing, analytes, plate, or person, but also due to the inherently varying nature of the capture probes (such as proteins) themselves. While fluorescent labeling provides sensitive detection after the assay incubation and labeling, it does not allow for determining the amount and the morphology of the capture probe spots prior to the experiment. In general, post image processing tools are utilized to improve the quality of DNA microarrays, or intermediate labeling steps are utilized for visualization of the printed slides prior to incubation [21]. IRIS, with its high-throughput quantification capability, provides an excellent platform for microarray quality control if the microarrays are developed on appropriate substrates. The basic requirement for the substrate is to have a layered structure (such as an oxide layer on Si substrates) to allow for the interferometric signature. It has been shown that Si chips are compatible with fluorescence assays and they provide considerable advantages by enhancing the fluorescence signal [22], thus allowing for a pre-calibration and quality control by IRIS. In light of these advantages, the utility of a calibrated fluorescence enhancement (CaFE) method has been demonstrated in [14,15,23] on an integrated platform. Significant improvements have been demonstrated for a particular application involving allergen-specific IgE detection with two major allergens, peanut (Ara h1) and timothy grass (Phl p1), whose immobilization can vary significantly making fluorescence signal calibration vital to obtain accurate results [17].

### 4.5. Single Particle Interferometric Reflectance Imaging Sensor

**Figure 7:**
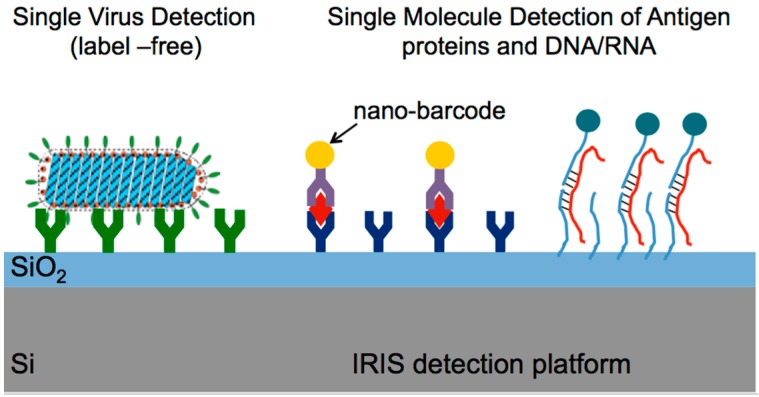
Concept of the biodetection assay for digital detection and counting of individual viral pathogens and antigens decorated with AuNP. (Reused with permission from [12]).

SP-IRIS, as a biosensing platform, builds on the established strength and versatility of antibody-based assays and adds a simple, but powerful, optical enhancement that allows the direct visualization of natural and artificial nano-particles captured on a layered Si chip as described above. With this method, particles that were previously too small to be detected by standard microscopy (e.g., viruses) can now be directly visualized, counted, and sorted by size and shape for diagnostic confidence [8]. Similarly, individual proteins labeled with small metallic nanoparticles can be counted on the sensor surface (as conceptually shown in Figure 7) for attomolar-level sensitive detection. The sensitivity of the IRIS platform has been dramatically increased with the introduction of single particle detection modality enabling digital sensing of nanoscale particles and viruses [24]. Below, we describe results on detection of individual viral pathogens and single protein counting with gold (Au) nanoparticle labels.

#### 4.5.1. Digital Label-Free Detection of Viruses

**Figure 8:**
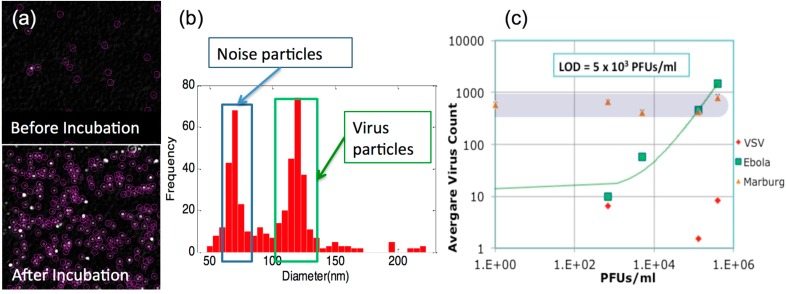
In virus detection experiments, by comparing particles found within the expected virus size range for pre- and post-incubation images (**a**) bound on the specific antibody spot, individual virions can be identified and counted. A histogram of virus sizes (**b**) is identified in each image. A size filter is chosen to remove background (noise) particles. (**c**) A typical dilution curve for Ebola VSV pseudotype concentration is shown with an LOD of 5 × 10^3^ pfu/mL in serum containing other viruses and bacteria (10^6^ cfu/mL *E. coli* K12 and 10^5^ pfu/mL of Marburg VSV pseudotypes). (Reused with permission from [12]).

Application of SP-IRIS for rapid diagnostics have focused on viral hemorrhagic fevers (Ebola, Marburg, and Lassa Fever). Detection and identification of virus particles in complex samples for replication-competent wild-type vesicular stomatitis virus (VSV), defective VSV, and Ebola- and Marburg-pseudotyped VSV have been demonstrated with high sensitivity and specificity in [25]. In that study, SP-IRIS chips were incubated with serum or whole blood spiked with virus titrations and later washed, dried, and read on the instrument. Size discrimination of the imaged nano-particles (virions) facilitates elimination of nonspecifically bound particles to achieve an LOD of 5 × 10^3^ PFU/mL (<10 atto-molar of viable virus) for the Ebola and Marburg VSV pseudotypes as shown in Figure 8.

The LOD for SP-IRIS is calculated from the mean plus three standard deviations of the control spots as explained in [25]. Simultaneous detection of multiple viruses in a single sample (serum, plasma, or whole blood) for screening applications, as well as uncompromised detection capabilities in samples contaminated with high levels of bacteria, have been shown. Both single and dual virus samples were made in serum containing 10^6^ CFU/mL *E. coli* K12.

#### 4.5.2. Multiplexed Protein Detection with Au Nanoparticle Labels

Since individual proteins are too small to be identified as particles, SP-IRIS utilizes nanoparticles as labels for “digital” detection. Such an approach is commonly referred to as “mass-tagging” and is a widely used method to enhance sensitivity. However, typical mass tagging requires micron-size particles resulting in limitations due to diffusion and steric hindrance caused by the large particle [26,27]. In contrast, SP-IRIS can detect Au nanoparticles as small as 20 nm, which is only about twice the hydrodynamic diameter of an antibody.

**Figure 9:**
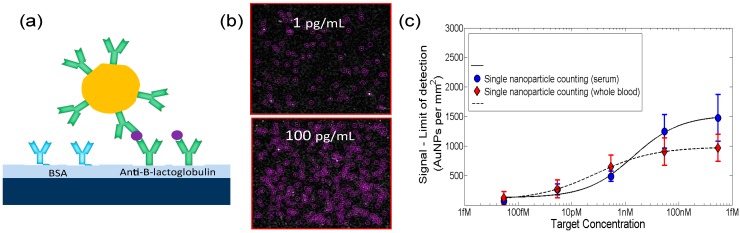
(**a**) Conceptual representation of SP-IRIS single molecule counting assay for protein biomarker detection; (**b**) Representative images at 1 pg/mL and 100 pg/mL target concentration; (**c**) Dilution curve for β-lactoglobulin in unprocessed serum and whole blood shown in blue and red, respectively. (Reused with permission from [28]).

Using this approach, detection of a protein biomarker, β-lactoglobulin, in both unprocessed serum and human whole blood has been demonstrated in [28]. Figure 9 shows the concept and a dilution curve for β-lactoglobulin incubated for 2 h in serum and in whole blood with a detection limit of 60 aM and 500 aM, respectively. Direct detection of protein biomarkers with attomolar sensitivity without the need of sample preparation can pave the way for performing diagnostic tests at the point of care. Furthermore, quantitation of allergen-specific IgE from unprocessed finger prick volume of human blood has also been shown in [28].

## 5. Alternative Techniques and Performance Comparisons

The development of label-free detection methods have enabled the study of primary interactions between biomolecules, such as antigen-antibody and protein-DNA, without requiring secondary probes binding to target analytes for a quantifiable signal [4]. ELISA, one of the most widely used immunoassays, is a label-based technique that makes use of the enzyme-linked secondary reagents to obtain a quantifiable signal [14]. The main disadvantage of this type of secondary probe-based biosensors is the complexity of the immunoassay due to its need for multiple binding layers. A critical innovative aspect of the IRIS platform is its ability to detect both biomasses and nanoscale particles in a label-free manner with its two distinct modalities.

As discussed earlier, SP-IRIS enables the label-free detection of individual virions captured onto the sensor surface overcoming a host of unique challenges due to their small size, low refractive index and polydispersity. Surface plasmon resonance (SPR) has been the leading technology among label-free detection methods [29], but for single particle detection, it has shown limited success partially due to the inherent roughness of the Au surface utilized in SPR. Other label-free optical biodetection technologies have generated interest with their simple and high-throughput operation as well; in particular, BIND (biomolecular interaction detector), utilizing diffraction gratings, has been applied to single particle detection [30]. In addition, resonant optical cavities, such as whispering gallery mode (WGM) sensors, as explained in [31], have also shown single particle detection capability. However, these frequency-shift-based sensors lack robust particle detection capacity, as their signal is highly dependent upon the binding position of the nanoparticle with regards to the evanescent tail of the optical wave on the resonator [9]. Thus, the aforementioned complex and delicate optical sensor systems have yet to show the robust single particle detection and analysis capability with size discrimination in complex biological fluids.

Akin to SP-IRIS, other interferometric nanoparticle detection methods have also been developed over the years. These include a method implementing confocal microscopy in an interferometric scheme using supercontinuum laser illumination [32], interferometric detection of scattering (iSCAT) [33], and another method implementing a background-free, interferometric scattering measurement of nanoparticles using a split detector [34]. All of the aforementioned interferometric techniques use a laser as a light source, which, owing to its highly coherent nature, results in speckles in the images, requiring further optical tools to overcome, as the speckles contribute to reduced signal-to-noise ratio. Furthermore, [32] requires sample scanning in a complex confocal setup, making it a costly alternative with no multiplexed detection capability, and [33] employs laser beam scanning using acusto-optic deflectors, which makes the detection platform expensive and very limited in terms of high-throughput.

**Table 1:** Performance comparisons for the IRIS platform and its alternatives in terms of the limit of detection.

Biosensor	Analyte	Limit of Detection	Reference
***SP-IRIS***	Protein (β-lactoglobulin)	60 aM	[28]
	Virus (Ebola-pseudotyped VSV)	5 × 10^3^ pfu/mL (<10 aM)	[25]
IRIS *	Protein (rabbit-IgG)	19 ng/mL (~120 pM)	[4]
	Protein (BSA)	5.2 pg/mm^2^	[14]
	Virus (VSV)	3.5 × 10^5^ pfu/mL	[7]
***iSCAT***	Protein (IgG1)	1 ng/mL (~6 pM)	[35]
***WGM***	Protein	0.03 pg/mm^2^	[31]
	Virus (InfA)	10 fM	[36]
***SPRi***	Protein (β_2_m/cysC)	1 nM	[37]
	DNA	10 nM	[38]

* The low-magnification biomass detection modality of the IRIS platform is referred to as SRIB (Spectra Reflectance Imaging Biosensor) in [4].

In Table 1, we provide a performance summary in terms of the limit of detection of the IRIS platform and other widely used alternative techniques as previously mentioned.

As can be seen from Table 1, the label-free biomass detection and digital single particle detection modalities together make the IRIS a biosensing platform with an unprecedentedly wide dynamic range in comparison with its alternatives [28].

## 6. Conclusions

For clinical applications, there is a need for diagnostics tools with sensitivity compared to existing state-of-the-art technologies without complicated assays, sample preparation, and bulky equipment. With the IRIS platform reviewed here, we have demonstrated detection of diagnostics targets of clinically relevant units from complex samples, with sensitivities comparable to laboratory assays, but in a much simpler format. IRIS offers detection of a variety of targets such as proteins, nucleic acids, and whole viruses in a simple assay format and with high sensitivity (at single pathogen or molecule resolution). In addition, its compact design, robustness, and low cost make the platform a promising tool for point-of-care assays. Through integration with microfluidics, IRIS may be implemented for critical point-of-care applications such as detection of biomarkers from unprocessed blood in primary care setting for early diagnosis or sample-to-answer virus detection in resource-poor rural settings for infectious disease control and containment during an outbreak.
